# An Apology to the Editor of the *Digest*

**Published:** 1896-04

**Authors:** 


### AN APOLOGY TO THE EDITOR OF THE "DIGEST."

Under the head of "Remarkable Misquotation" in the February number of the *Dental Digest*, the editor, Dr. Crouse, very properly takes the editor of this journal to task for misquoting him in the editorial entitled, "The War on Dental Colleges." It is needless to say that we endeavor to be correct always in quoting the language of another, not only that we may do no injustice, but for our own protection. An examination of Dr. Brown's paper abundantly proves that we were in error in this instance, and that Dr. Crouse had excellent reasons for his strictures. We regret the mistake, which was made by a too hurried examination of the manuscripts without proper subsequent reference to the printed page. In order that justice may be done Dr. Crouse, we quote from the *Digest* his remarks upon the subject in dispute: "A young man may be bright, a good student, and well-grounded in the classics, and yet an attempt to make a dentist of him would destroy his usefulness in life, make him a detriment to the community in which he practised, and not a credit to the dental profession. Therefore, the first six weeks of the college course should be spent in finding out who are properly qualified by nature, as well as by training, to be dentists, and those who are not fit should have their fees refunded, and should be persuaded to select a more suitable calling in life. In short, the 'plucking' should be done at the beginning of the college course rather than at the end."

Now, the expression quoted which seems to have roused our friend seems, to our comprehension, very near to his own idea. Credit should have been given Dr. Brown, the writer of the paper on "Dental Examining Boards and Preliminary Education," in which he says, "Now, as to a student's mechanical adaptability: granted a first-class education and the receptive qualities necessary for a good scientific education, of what avail is it 'if he hasn't it in his fingers,' as Dr. Eaton has so tersely expressed it? he will never make a dentist, and of this fact the colleges have as yet taken no notice." There seems to be a perfect unison of sentiment in these two quotations.

We do not care to follow the editor of the *Digest* in his criticisms of the editorial in the February number of this Journal. Those who have had a lengthened experience in college work are alone entitled to speak on the duties of deans, and we do not regard the editor of the *Digest* as a competent witness. A third of a century of practical college labor has demonstrated to our comprehension that the views set forth in the editorial alluded to are correct; but if we are called in the future to revise our opinions, we will endeavor to seek light from some other quarter than the office of the *Digest.*

This constant iteration of what colleges and deans of colleges should do or leave undone, by persons having no practical experience, reminds us of a group of children theorizing as to what they will do when they become men and women. Experience will have demonstrated in after years that practical knowledge is very destructive to "castles in the air."

It is one thing to have the experience of a student, another that of a teacher, and still another that of the dean. Each of these positions gives special facilities for observation and practical knowledge, and we kindly recommend the editor of the *Digest* to acquire wisdom by a living experience, or, failing in the opportunity for this, secure a consensus of the opinions of those who have had the management of colleges for a decade or more, and we feel sure that they will agree with us that it is quite impossible to find out "in the first six weeks of college life" the natural bent of a young man. Had this judgment been exercised with some, now dentists of repute, we fear the writer of this would have made a serious mistake. There is, probably, no more embarrassing position for a young man without previous experience than a first year in a dental college, and he requires the kindly care of those intrusted with his education, and not crude criticism that may be very unjust. There is no more important study than the possible capabilities of young men, and, in our opinion, the man who imagines he can master this in six weeks, or even in a year, has a very poor conception of the difficulties of the task he has set himself to conquer.

